# Low-density lipoprotein receptor-related protein-1 (LRP1) in the glial lineage modulates neuronal excitability

**DOI:** 10.3389/fnetp.2023.1190240

**Published:** 2023-06-13

**Authors:** Andreas Faissner

**Affiliations:** Department of Cell Morphology and Molecular Neurobiology, Ruhr University Bochum, Bochum, Germany

**Keywords:** astrocyte, Emx1, epilepsy, low-density lipoprotein receptor-related protein-1 (LRP1), NG2 (CSPG4) chondroitin sulfate proteoglycan, radial glia, tissue plasminogen activator (tPA), (GLAST) glutamate aspartate transporter

## Abstract

The low-density lipoprotein related protein receptor 1 (LRP1), also known as CD91 or α-Macroglobulin-receptor, is a transmembrane receptor that interacts with more than 40 known ligands. It plays an important biological role as receptor of morphogens, extracellular matrix molecules, cytokines, proteases, protease inhibitors and pathogens. In the CNS, it has primarily been studied as a receptor and clearance agent of pathogenic factors such as Aβ-peptide and, lately, Tau protein that is relevant for tissue homeostasis and protection against neurodegenerative processes. Recently, it was found that LRP1 expresses the Lewis-X (Lex) carbohydrate motif and is expressed in the neural stem cell compartment. The removal of *Lrp1* from the cortical radial glia compartment generates a strong phenotype with severe motor deficits, seizures and a reduced life span. The present review discusses approaches that have been taken to address the neurodevelopmental significance of LRP1 by creating novel, lineage-specific constitutive or conditional knockout mouse lines. Deficits in the stem cell compartment may be at the root of severe CNS pathologies.

## 1 Introduction

### 1.1 Identification of Lewis-X carrying glycoproteins in radial glia

Radial glia cells represent the principal neural stem and glial progenitor cell (NSPC) compartment during neural development (Malatesta and Gotz, 2013). The development of the CNS is a carefully regulated process where neurogenesis precedes the formation of oligodendrocytes and astrocytes (gliogenesis) (Gotz and Huttner, 2005; Kriegstein and Alvarez-Buylla, 2009; Taverna et al., 2014). Gliogenesis begins around embryonic day (E) 12.5 in the mouse CNS with the specification of oligodendrocyte precursor cells (OPCs) (Bergles and Richardson, 2016). Astrocytes represent an abundant glial population of the nervous system of mammals that emerges at later stages and displays a considerable heterogeneity that has so far not been appreciated (Allen and Barres, 2009; Zhang and Barres, 2010; Clarke and Barres, 2013; Molofsky et al., 2014). Despite their various roles in CNS function and homeostasis their ontogenetic development remains poorly understood (Faissner et al., 2010; Molofsky et al., 2012). Increasing evidence suggests that the extracellular matrix (ECM) microenvironment of the stem cell niche plays an important role for the differentiation of neural stem and glial progenitor cells (Garcion et al., 2004; Faissner and Reinhard, 2015; May et al., 2018; Kjell et al., 2020; Schaberg et al., 2022). In this regard, we have addressed novel glycan markers of the neural stem cell compartment established in our laboratory, in particular LewisX related epitopes (Hennen et al., 2011; Hennen et al., 2013). LewisX (LeX), also known as CD15 or stage specific embryonic antigen-1 (SSEA-1), is a carbohydrate motif associated with glycoproteins and glycolipids. Throughout neurogenesis, LeX is detectable on radial glia that represents the major NSPC population in the developing embryonic CNS (Hennen and Faissner, 2012). Using novel monoclonal antibodies we revealed a considerable microheterogeneity of LeX-related glycans that display differential expression patterns on glial surfaces (Hennen et al., 2011). The LeX-epitopes recognized by the monoclonal antibodies 487^LeX^ and 5750^LeX^ we characterized are shared to some extent by radial glia subpopulations and hiPSC-derived stem cells and partially sensitive to proteolysis (Kandasamy et al., 2017; Roll et al., 2022). In an effort to resolve the apparent heterogeneity of glial surface expression, we characterized LeX-expressing protein cores in the CNS and identified Phosphacan, a secreted splice variant of the protein tyrosine phosphatase receptor-type zeta (Ptprζ1) (Garwood et al., 1999), the ECM glycoprotein tenascin-C (Faissner et al., 2017), the L1-cell adhesion molecule (L1-CAM), and, finally, the lipoprotein receptor-related protein 1 (LRP1) as a novel carrier protein (Hennen et al., 2013). The β1-integrin, lysosomal-associated membrane protein (LAMP-1), CD24 and Thy-1 are further known LeX carriers (Hennen and Faissner, 2012).

### 1.2 LRP1 is a scavenger expressed by NSPCs and their progeny

**FIGURE 1:**
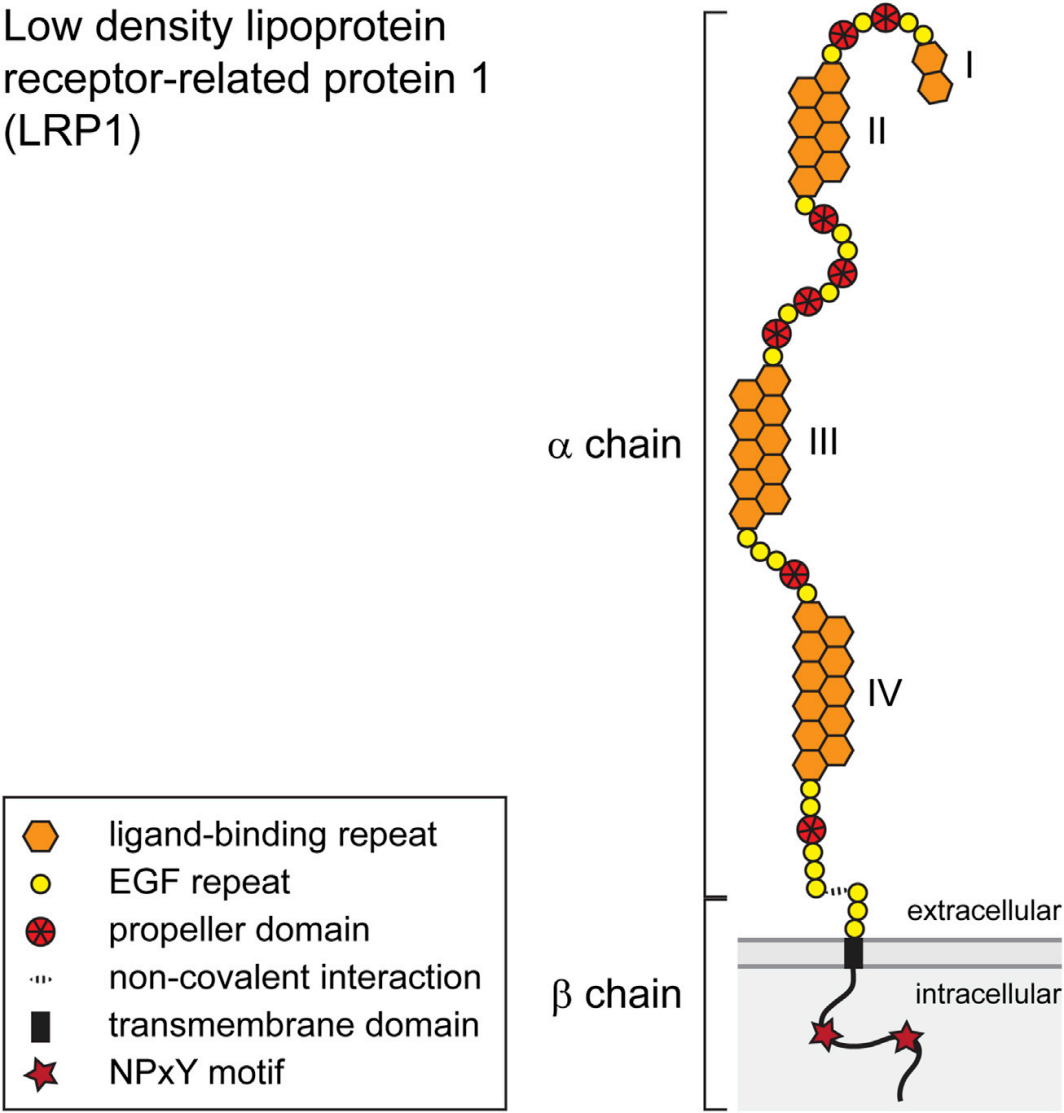
Schematic representation of the LRP1 scavenging receptor. The single pass transmembrane receptor protein consists of a 515 kD extracellular α-chain and a transmembrane β-chain of 85 kD which are connected via non-covalent interactions. Both chains are generated from a single-chain precursor protein by a furin-type protease in the Golgi apparatus. The α-chain is characterized by repeat domains including egf-type, egf-precursor-protein type, β-propeller-type repeats and cysteine-rich ligand binding domains I–IV. The intracellular segment of the β-subunit possesses two NPxY motifs and one YxxL motif and two di-leucine motifs. The β-subunit furthermore contains docking sites for PSD-95, Dab-1 and FE-65. For details see text.

LRP1, also known as CD91, is member of the LDL-family of receptors and synthesized as a type 1 transmembrane receptor with 4,525 amino acids that is cleaved by a furin-type protease in the Golgi apparatus (Herz et al., 1988). The cleavage produces an extracellular α-chain of 515 kD and a transmembrane β-chain component of 85 kD which stay both connected by non-covalent interaction (Boucher and Herz, 2011). The structure of LRP1 comprises cysteine-rich egf-type repeats, β-propeller domains, a transmembrane domain and an intracellular domain that contains the NPxY motif (Herz and Strickland, 2001) (Figure 1).

**FIGURE 2:**
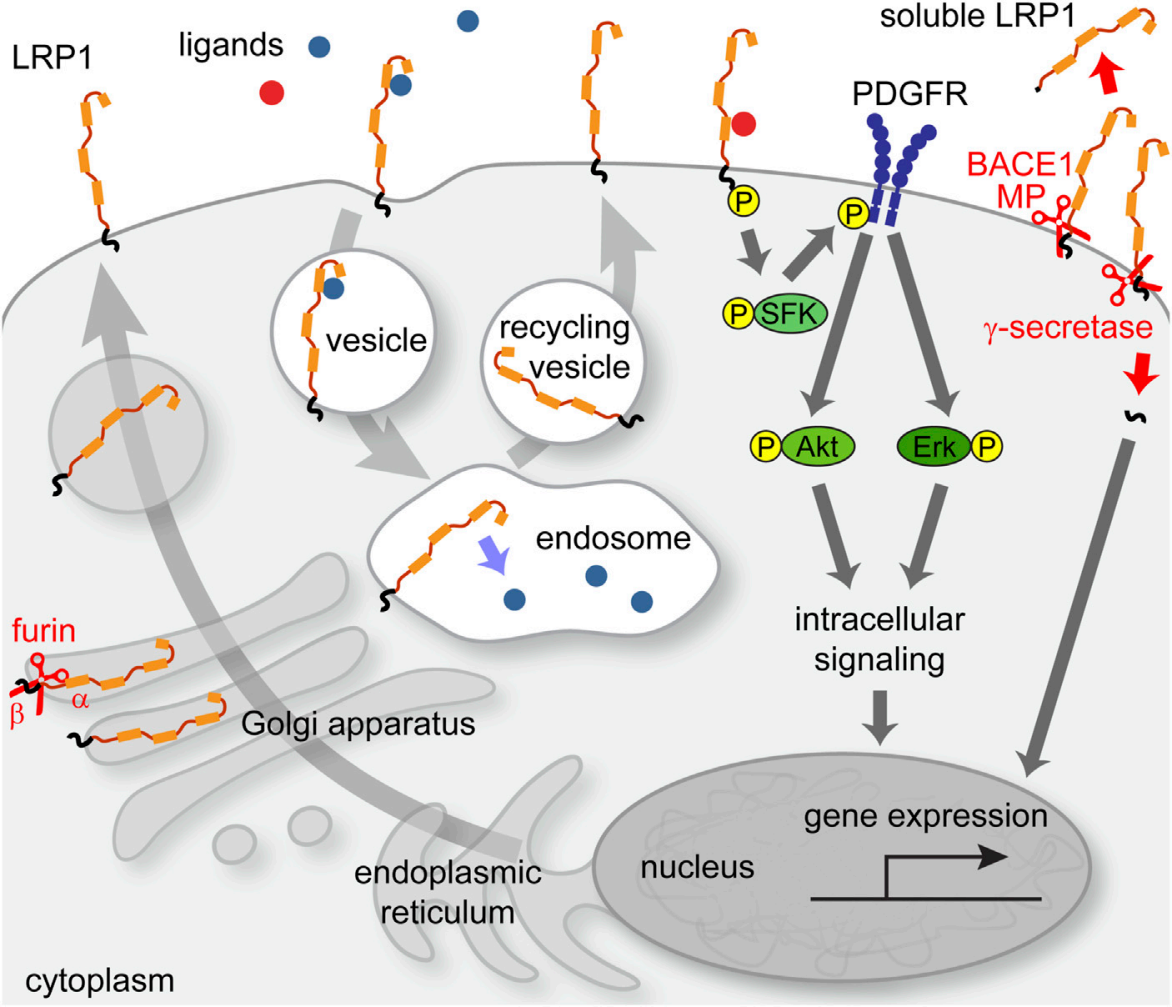
Intracellular pathways of LRP1 receptor. LRP1 precursor protein is cleaved by a furin-type protease in the Golgi apparatus and transported to the membrane. Upon ligand binding, an endocytosis process is initiated that leads to transit of LRP1 to the endosomal compartment, from where it is recycled back to the membrane after the release of cargo. In association with other receptors, e.g., PDGFRβ, LRP1 can elicit signal transduction pathways that can impact cellular functions such as gene regulation via the Erk-pathway.

LRP1 comprises ligand binding sites for more than 40 binding partners, including ApoE, proteases, protease inhibitors, extracellular matrix components, cytokines, growth factors, bacteria and viruses (Lillis et al., 2008; Boucher and Herz, 2011; Bres and Faissner, 2019b). Its primary function is linked to processes of receptor-mediated endocytosis of ligands, whereupon LRP1 is recycled to the cell membrane surface (Figure 2). A receptor associated protein (RAP) has been discovered that binds to the ligand-binding regions and thereby blocks any receptor-cargo interactions (Bu et al., 1994). The LDL-family of receptors and its member LRP1 have classically been associated with a modulation of lipoprotein metabolism and have been studied for example, with reference to Alzheimer’s disease (Hussain et al., 1999; Jaeger and Pietrzik, 2008; Eggert et al., 2018; Sousa et al., 2023). In addition, diverse functions for this receptor have been uncovered in various aspects of cellular activities, including cell proliferation, migration, differentiation, immune response and survival (Herz and Bock, 2002; Sizova et al., 2023).

Interestingly, the α-subunit can be released into the extracellular space where it exerts functions independently of the β-subunit. Soluble sLRP1 is the major transport protein for Abeta and prevents the re-entry of the circulating protein from the plasma into the brain (Sagare et al., 2012). The β-subunit that persists in the membrane is cleaved by γ-secretase and the resulting fragment can transit to the nucleus and influence gene transcription (May et al., 2002; Zurhove et al., 2008). In the CNS, LRP1 is expressed by neuronal and glia cell populations (Auderset et al., 2016a). Furthermore, the LRP1 receptor is prominent in the blood-brain-barrier (BBB) where it is present in endothelia, astrocytic end-feet, pericytes and smooth muscle cells (Ma et al., 2018; Storck et al., 2021b; Oue et al., 2023). Interestingly, LRP1 is also expressed in the neural stem cell compartment, by NSPCs (Hennen et al., 2013; Auderset et al., 2016b). However, its potential roles during neural development had not been addressed so far. In order to study its functions in the radial glia stem cell and glial progenitor compartments, we have exploited the *Lrp1* ^*fl/fl*^ mouse line and deleted the receptor by means of Cre-loxP mediated recombination *in vitro* using recombinant membrane permeable Cre-recombinase (Nolden et al., 2006). The functional status of LRP1-deficient cells was studied using proliferation, differentiation and apoptosis assays. LRP1-deficient NSPCs from both embryonic spinal cord and cortex demonstrated an altered differentiation potential. The differentiation capacity towards oligodendrocyte precursor cells (OPCs), mature oligodendrocytes and neurons was reduced. In contrast, astrogliogenesis was promoted. Moreover, *Lrp1* deletion had a negative effect on NSPC proliferation and survival. Our observations suggested that LRP1 facilitates NSPC differentiation via interaction with ApoE. Upon ApoE4 stimulation wild type NSPCs generated more oligodendrocytes, but LRP1 knockout cells showed no response. The effect of ApoE4 seemed to be independent of cholesterol uptake, but was rather mediated by downstream MAPK and PI3K/Akt activation. In conclusion, in the light of our results LRP1 was characterized as a membrane receptor that controls important signalling pathways in the glial stem and progenitor cell compartment of the developing CNS (Hennen et al., 2013; Safina et al., 2016).

### 1.3 *Emx1* as driver of conditional *Lrp1* knockout

In light of the functional roles of LRP1 in the radial glia stem and glial progenitor cell compartments, we decided to investigate its roles in neural stem cells *in vivo*. To this end, we created a mouse line by crossing the *Lrp1* ^*fl/fl*^ animals with an *Emx1-Cre* driver line (Table 1). The mouse empty spiracles homeobox gene family consists of the members *Emx1* and empty spiracles homeobox 2 (*Emx2*). Both regulatory genes are restricted to the forebrain. The *Emx1* gene expression in the mouse starts at E9.5 and is confined to the dorsal forebrain that gives rise to the cerebral cortex, olfactory bulbs and hippocampi. The *Emx1* gene is found in radial glia, postmitotic projection neurons of the dorsal, lateral and medial pallium forming the neocortex, piriform cortex and hippocampi, respectively, as well as in astrocytes and oligodendrocytes of the pallial corpus callosum and fimbria in the developing brain (Cecchi and Boncinelli, 2000; Gorski et al., 2002). The *Emx1-Cre::Lrp1* ^*fl/fl*^ line drives the expression of the Cre-recombinase in the telencephalic radial glia compartment and thereby allows for a selective elimination of a gene of interest in the radial glia cells of the forebrain (Iwasato et al., 2000). We obtained viable offspring where the *Lrp1* gene had selectively been deleted in the cortical radial glia lineage (Table 1). *Emx1* lineage cells do not give rise to GABAergic inhibitory interneurons and subpallial oligodendrocytes. Considering the *Emx1Cre* distribution, it was expected that in the novel mouse mutant the *Lrp1* gene would only be absent in dorsal forebrain radial glia and their progeny. However, the *Emx1* expressing cells located in the basal telencephalon can form two distinct populations. The first one gives rise to pyramidal-like, burst-firing, excitatory cells of the amygdala while the second population migrates from the pallium to the subpallium and finally differentiates into medium spiny neurons (Cocas et al., 2009).

**TABLE 1:** Conditional and constitutive mouse Lrp1 knock-out lines.

Promoter target	Reporter	Mouse line	*In vitro* phenotype	*In vivo* phenotype	References
Emx1 Cortical RG	None	*Emx1-Cre::Lrp1* ^*fl/fl*^	• Reduced uptake of tPA in astrocytes	• Seizures, Motor deficits	Bres et al. (2020)
				• GFAP elevated	
				• c-Fos elevated	
				• Altered tPA	
				• Postnatal lethal (70%)	
GFAP Astrocytes	None	*GFAP-Cre::Lrp1* ^*fl/fl*^	• Decreased Aβ42 uptake	• Amyloid deposition up	Liu et al. (2017a)
			• Reduced level of proteases	• Reactive astrocytes up	
				• Release inflammatory Cytokines	
GLAST Astrocytes	tdTomato	*GLAST-CreER* ^*T2*^ *::Lrp1* ^*fl/fl*^	• Astrocyte maturation delayed	• Astrocyte maturation delayed	Romeo et al. (2020), Romeo et al. (2021)
			• Changes in coculture	• Activated Erk down	
			• Synaptogenesis changed	• cFos reduced	
			• Neural network activity down		
			• Cytokines altered		
NG2 NG2-cells	eGFP	*NG2-CreER* ^*T2*^ *::Lrp1* ^*fl/fl*^	• Vulnerability of OPCs up	• Progressive loss of OPCs	Schäfer et al. (2019)
			• Fibre myelination changed		
CAGOPCSOlig2 PDGFRα	None	*CAG-CreER* ^*TM*^ *::Lrp1* ^*fl/fl*^	• OPC cholesterol content down	• WM repair attenuated	Lin et al. (2017)
		*Olig2-Cre::Lrp1* ^*fl/fl*^ *PDGFRα-Cre::Lrp1* ^*fl/fl*^	• Myelin sheets down	• OPC differentiation down	
			• SREBP in OPCs up	• Reduced myelination of ON	
				• Peroxisomes impaired	
PDGFRα OPCs	YFP mGFP	*PDGFRα-Cre::Lrp1* ^*fl/fl*^	• OPC differentiation up	• In adult OPC prolif. up	Auderset et al. (2020)
				• In adult myelination up	
				• Cuprizone lesion repair up	
CaMKII Neurons	None	*CaMKII-Cre::Lrp1* ^*fl/fl*^	• Cholesterol in neurons down	• Lipid metabolism changed	Liu et al. (2010)
				• Changes of spines	
				• Neurobehavioral deficits	
CaMKII Neurons	None	*CaMKII-Cre::Lrp1* ^*fl/fl*^		• Aβ−plaques up	Tachibana et al. (2019)
				• Aβ in hippocampus up	
Synapsin Neurons	None	*Synapsin-Cre::Lrp1* ^*fl/fl*^	• Colocalization of LRP1 and NR2A	• Motor deficits	May et al. (2004)
				• Behavioural deficits	
				• Reduced life span	
Nestin Neural precursors	None	*Nestin-Cre::Lrp1* ^*fl/fl*^	• Reduced neurite branching		Nakajima et al. (2013)
			• PSD-95 degradation down		
			• GluA1 internalization down		
Tie2 Endothelial cells	None	*Tie2-Cre::Lrp1* ^*fl/fl*^		• BBB breakdown	Nikolakopoulou et al. (2021)
				• Neuron loss	
				• Cognitive deficits	
Slco1c1 CNS endothelium	None	*Slco1c1-CreER* ^*T2*^ *::Lrp1* ^*fl/fl*^	• Reduction of claudin and tight junction in endothelia	• Loss of BBB integrity	Storck et al. (2021b)
			• MMP enhanced	• Tight junction degradation	
				• P-glycoprotein reduction	
sm22α Vascular smooth muscle cells	None	*Sm22α-Cre::Lrp1* ^*fl/fl*^		• Loss of BBB	Oue et al. (2023)
				• Cognitive impairment	
Cspg4 Pericytes, NG2 glia	None	*Cspg4-Cre::Lrp1* ^*fl/fl*^	• Clearance of Abeta *in vitro* reduced		Ma et al. (2018)
			• Clearance by capillaries in brain explants reduced		

### 1.4 Elimination of *Lrp1* from radial glia causes and epileptic seizures

Epilepsy is a neurological disorder that affects up to 1% of the population and leads to severe restrictions of the private and professional lives of those affected (Binder and Steinhauser, 2006). A large range of conditions and pathophysiological developments can cause the disease that is characterized by a shift of the balance between excitation and inhibition (Devinsky et al., 2013; Patel et al., 2019). This imbalance eventually leads to hyperexcitability in distinct brain regions (Stafstrom, 2006; Fritschy, 2008; Steinhäuser and Seifert, 2012; Devinsky et al., 2018; Righes Marafiga et al., 2021). A quest for molecular mechanisms may identify novel, potentially interesting genes as novel targets for therapeutical intervention (Goldberg and Coulter, 2013). In the *Emx1-Cre::Lrp1* ^*fl/fl*^ knockout mouse pronounced, generalized seizure episodes occurred spontaneously as early as at postnatal day P19 (Bres et al., 2020). An analysis conducted with the c-fos marker revealed that hyperactive neurons were located in the hippocampus, in deep cortical layers and in the thalamus. It is plausible that epileptic activity beginning in the dorsal telencephalon can spread to basal ganglia and higher-order brain regions, resembling the c-fos expression pattern (Constantinople and Bruno, 2013). We have not been able to localize a distinct region of origin of the seizure activity, which would require sophisticated EEG-recording techniques with higher resolution. In a subpopulation of knockout mice the seizures recurred daily and were accompanied by deteriorating health including decreased body weight and, finally, a reduced life span. Indeed, more than 70% of the affected animals did not survive beyond 3 months (Bres et al., 2020). The severity of the phenotype was unexpected and contrasts investigations concerning the role of LRP1 in the neuronal lineage. In fact, ablation of *Lrp1* in neurons using *CaMKII-Cre* mouse lines yielded solely symptoms of hyperexcitability that did not, however, progress to seizure-like symptoms (May et al., 2004; Liu et al., 2010; Maier et al., 2013).

In addition to the presence of seizures, *Emx1-Cre::Lrp1* ^*fl/fl*^ mice exhibit a clasping reflex when held by the tail. As motor-coordination deficits were detected previously in *Lrp1* neuronal knock-out mice (Liu et al., 2010) behavioral tests were performed. The results of a panel of motor tests including the rotarod test, the hangwire test and footprint analysis confirmed that *Emx1-Cre::Lrp1* ^*fl/fl*^ knock-out mice suffered from cortically induced ataxia (Bres et al., 2020). The cortical circuitry resulting in ataxia in the *Emx1-Cre::Lrp1* ^*fl/fl*^ line has not been elucidated in detail. The *Emx1* lineage medium spiny neurons contribute both to movement facilitation and inhibition, but the majority is involved in movement inhibition (Cocas et al., 2009). In this regard, *Emx1*-expressing medium spiny neurons which receive dopaminergic input from and provide output to the substantia nigra may contribute to the effect. The prefrontal cortex and the nigrostriatal system are involved in movement initiation and the control of motor activity (Laruelle et al., 2003).

In the *Emx1-Cre::Lrp1* ^*fl/fl*^ mouse mutant both neuron density and cortical layering were not affected. In this respect the model is similar to the *synapsin-Cre::Lrp1* ^*fl/fl*^ mutant described earlier (May et al., 2004). Neuron maturation and viability were also not compromised in the *Nestin-Cre::Lrp1* ^*fl/fl*^ mouse, but reduced neurite growth and branching was observed (Nakajima et al., 2013). This observation corresponds to the reduced length of neurites growing out from *Lrp1*-deficient neural stem cells *in vitro* (Safina et al., 2016). Along these lines, blockade of LRP1 by RAP also impaired neurite outgrowth (Qiu et al., 2004). In a neuronal knockout of *Lrp1* driven by *CaMKII-Cre* a reduction of spines has been reported in cortical and in hippocampal neurons (Liu et al., 2010).

The observation of seizures in the *Emx1-Cre::Lrp1* ^*fl/fl*^ mouse model raises the question whether LRP1 mutations may be a cause of seizures in the human. To date, no reports are available that support an involvement of LRP1 in human epilepsy. However, genetic studies suggest a link between LRP1 truncation variants and autism and pleiotropic effects across psychiatric phenotypes (Torrico et al., 2019). With regard to epilepsy, an enhanced expression of LRP1 was detected in foci of a kainate mouse model of drug-resistant epilepsy (DRE). On that basis, a micellar-based LRP1-targeted paramagnetic Gd^3+^-LP probe was developed to visualize epileptic foci *in vivo*. This approach was efficient and informative both in the mouse model and in human DRE patients (Wang et al., 2021). This observation allowed for the application of the MRI probe to circumscribe epileptic foci in the context of therapeutic surgery (Li et al., 2023). This represents an application of the upregulation of LRP1 in seizure territories.

### 1.5 Ventricular enlargement in the *Emx1-Cre::Lrp1* ^*fl/fl*^ mouse

A striking phenotype characteristic found in P28 *Emx1-Cre::Lrp1* ^*fl/fl*^ mice is the enlargement of the volume of the lateral ventricles. Using the magnetic resonance imaging (MRI) technique, it was ascertained that knock-out mice displayed a significant enlargement of the lateral ventricular volume and a compression of the dorsal hippocampus. Remarkably, the expansion of ventricular volume was only mildly visible at P14, but clearly evident after 4 weeks of age, in close correlation with the appearance of seizures and weight loss. The increased ventricular size may translate into the hippocampal compression observed in juvenile *Emx1-Cre::Lrp1* ^*fl/fl*^ mice. Several pathways may lead to this complex phenotype. One plausible cause may be the augmented production of cerebrospinal fluid (CBF). A possible imbalance of fluid production and removal in the ventricular space addresses the neurovascular unit (NVU). This structure consists of the endothelia that line the lumen of the capillaries, the basement membrane the endothelia appose to, the pericytes that cover the surface of the vessels and the astrocytic processes that abut to the external face of the blood vessel (Kaplan et al., 2020). The diameter of the vessels is in part regulated by vascular smooth muscle cells (VSMC) (Sweeney et al., 2016). These cellular components cooperate to form the blood-brain barrier (BBB) that protects the inner milieu of the CNS and regulates the exchange with the blood stream (Kaplan et al., 2020). Thus, astrocytic function may be disturbed, which has also been discussed as a potential reason for the pathophysiology of epilepsy and BBB integrity (Bedner et al., 2015; Robinson and Jackson, 2016; Liu et al., 2017a).

Astrocytic LRP1 and tPA regulate blood-brain barrier (BBB) permeability and inadequate tPA-LRP1, tPA-neuroserpin or tPA-Mac-1-LRP1-PDGFRβ interactions in the area of the BBB affect the permeability of the neurovascular unit in the context of seizures (Polavarapu et al., 2007; Niego et al., 2012; Nakajima et al., 2014; Fredriksson et al., 2015; Su et al., 2017). The increased ventricular space could reflect malfunctioning of the astrocytic membranes and perivascular astrocytic end feet. The aquaporin 4 (AQP4) transmembrane water channel protein is highly expressed by astrocytes and plays a pivotal role for extracellular space volume regulation and waste clearance by the glymphatic system (Haj-Yasein et al., 2011; Eidsvaag et al., 2017). AQP4 has been implicated in modifications of the BBB in the context of Alzheimer disease and temporal lobe epilepsy (Alvestad et al., 2013; Hubbard et al., 2016; Lan et al., 2017). In agreement with a role of AQP4, mice deficient for the channel suffer from hydrocephalus, accompanied by enlarged ventricles and elevated intracranial pressure (Verkman et al., 2006). Whether AQP4 is significantly changed in our model has not been addressed and is an interesting question for further studies (Bres et al., 2020).

Interestingly, LRP1 has been attributed a role in preventing over-proliferation of vascular smooth muscle cells which are important for blood vessel integrity (Boucher et al., 2003; Boucher et al., 2007; Boucher and Herz, 2011). Along these lines, PDGFRβ signaling intervenes in the recruitment of pericytes in the course of angiogenesis (Zhang et al., 2009) and deficits in blood vessel formation have been associated with temporal lobe epilepsy (Rigau et al., 2007). In this regard it is of interest that the interaction of tPA with PDGFRα is important for BBB integrity (Fredriksson et al., 2015). Because of the impact of the removal of LRP1 on the levels of tPA in the CNS potential modifications of the vascular system in the *Emx1-Cre::Lrp1* ^*fl/fl*^ mutant merit further investigations (Stefanitsch et al., 2015). Using genetic techniques, the roles of LRP1 expression in the cellular components of the NVU has been studied in more detail (Table 1). Using a *Tie2-Cre::Lrp1* ^*fl/fl*^ where LRP1 was eliminated in all endothelia the BBB was progressively degraded which resulted in neuronal loss and cognitive deficits (Nikolakopoulou et al., 2021). In an approach that was focused on the endothelia of the CNS the *Slco1c1-Cre::Lrp1* ^*fl/fl*^ similarly displayed a degradation of the BBB which could be traced to a degradation of the tight junctions of the endothelia and a reduction of the P-glycoprotein (Storck et al., 2021b). These observations are highly relevant in the context of Alzheimer disease, as LRP1 has been attributed an important role in the clearance of Abeta from the CNS (Storck et al., 2016; Storck and Pietrzik, 2017; Petrushanko et al., 2023).

Beyond the endothelia also the pericytes play important roles in the BBB. Thus, pericytes contribute to the integrity of the BBB, are involved in the regulation of angiogenesis, have phagocytic activities, contribute to CBF regulation and to neuroinflammatory responses (Sweeney et al., 2016). The ablation of LRP1 from pericytes in a *Cspg4-Cre::Lrp1* ^*fl/fl*^ mouse line documented that pericytes can clear Abeta peptide in an LRP1/ApoE-dependent mechanism (Ma et al., 2018). As a further component, the expression of LRP1 in VSMC has been tested in a genetic approach where cell-type restricted deletion was carried out in a *sm22α-Cre::Lrp1* ^*fl/fl*^ mouse line. The functions of LRP1 were assessed against an APOE3 or APOE4 background. In the latter, the removal of LRP1 led to impaired spatial memory, accompanied by a disruption of the BBB (Oue et al., 2023). These studies clearly emphasize that LRP1 plays important roles in the BBB and partakes in the clearance of Abeta (Cai et al., 2018; Eggert et al., 2018; Storck et al., 2021a; Petrushanko et al., 2023). In several instances the cell-type specific deletion of LRP1 resulted in compromised BBB function and, consequently, increased permeability of the BBB. Whether the BBB is compromised in the *Emx1-Cre::Lrp1* ^*fl/fl*^ model remains to be seen (Bres et al., 2020). In this context, it is of interest that Alzheimer disease is associated with seizures and epilepsy (Purushotham et al., 2022; van Vliet and Marchi, 2022; Yang et al., 2022). The potential association between BBB deficits, Alzheimer disease and seizures in the available mouse models will have to be explored further.

### 1.6 LRP1 and glutamate signaling

PSD-95 is a scaffolding protein of the membrane-associated guanylate kinase (MAGUK) that is highly enriched at the postsynaptic density. Among other structural features it comprises three conserved PDZ domains and one Src-homology 3-guanylate kinase module (Cheng et al., 2006). PSD-95 expression was found reduced at stages P28 and P56 both in the *Emx1-Cre::Lrp1* ^*fl/fl*^ and the *CaMKII-Cre::Lrp1* ^*fl/fl*^ mutants (Liu et al., 2010; Nakajima et al., 2013; Bres et al., 2020). PSD-95 interacts with a variety of receptors and proteins at the excitatory synapse and is implicated in the tuning of synaptic strength by regulating the number of alpha-amino-3-hydroxy-5-methyl-4-isoxazolepropionic acid (AMPA) receptors (May et al., 2004; Bats et al., 2007; Nakajima et al., 2013). PSD-95 is rather stable and serves to anchor AMPA receptors at the synapse (Chen et al., 2011). The reduction of PSD-95 leads to a reduction of the amount of AMPA receptors (Elias et al., 2006). Surprisingly, the elimination of PSD-95 does not severely affect synaptic transmission, probably because the loss is compensated by other MAGUK-family members (Elias et al., 2006; Chen et al., 2011).

The onset of epileptic seizures in the *Emx1-Cre::Lrp1* ^*fl/fl*^ mutant corresponds with the period of circuit maturation and maximum NMDAR expression (Szczurowska and Mareš, 2013). LRP1 interacts with PSD-95 and the NMDAR and thereby supposedly affects NMDAR-dependent calcium currents (Gotthardt et al., 2000; Qiu et al., 2002; Martin et al., 2008). It has been proposed that the hyperactivity and dystonia that occur in *Synapsin-Cre::Lrp1* ^*fl/fl*^ mutants are the consequence of missing modulation of ligand-dependent ion currents caused by the absence of LRP1 (May et al., 2004). In accordance with this assumption, the stimulation of neurons with N-Methyl-D-aspartate (NMDA) causes a reduction of PSD-95-LRP1 complexes, suggesting that LRP1 interacts with the active pool of PSD-95 which is degraded in response to NMDA treatment (Colledge et al., 2003; May et al., 2004).

A link between epilepsy and the downregulation of PSD-95 has been suspected (Wyneken et al., 2003; Sun et al., 2009). NMDARs are ionotropic glutamate channels and play a key role for excitatory synaptic transmission. These tetrameric receptors are composed by two obligatory GluN1 subunits, regulatory GluN2A-D and NMDAR (GluN3)A-B subunits. NMDARs are display high permeability for Ca^2+^, a voltage-dependent Mg^2+^ block and require the simultaneous binding of glutamate and d-serine or glycine for channel activation (Traynelis et al., 2010; Hansen et al., 2018). During development the NMDARs switch the subunit composition from GluN2B to preferentially GluN2A comprising combinations (Liu et al., 2004). GluN2A-containing receptors are mostly present at synapses whereas GluN2B are extrasynaptically located (Pal, 2018). In the *Emx1-Cre::Lrp1* ^*fl/fl*^ knock-out mice, no developmental changes of the obligatory NMDAR subunit GluN1 have been noted *in vivo* (Bres et al., 2020). However, the level of GluN1 was found increased in the membrane of NSPC-derived neurons *in vitro* using a cell surface biotinylation technique (Safina et al., 2016). Different from this result, cortical neurons obtained from E15-16 cortex of a *Nestin-Cre::Lrp1* ^*fl/fl*^ mouse cultured for up to 12 days did not reveal modifications of NMDAR levels (May et al., 2004). However, GluN1 expression was reported reduced in cortical neurons of a *CaMKII-Cre::Lrp1* ^*fl/fl*^ knockout mouse line (Liu et al., 2010; Gan et al., 2014). Along these lines a reduction of both GluN2B and the AMPA receptor subunits GluA1 and GluA2 was observed in a kainate rat model of epilepsy (Egbenya et al., 2018). These apparent differences may result from the differing promoters driving the Cre-recombinase.

In this regard, it is of interest to consider the opposing strategy, namely, the consequences of knock-in mediated overexpression. There, an upregulation of GluN1 and GluN2B in cultured neurons was noted which translated into behavioral modifications, such as hyperactivity and cognitive deficits (Roebroek et al., 2006; Reekmans et al., 2009; Maier et al., 2013). NMDAR expression is augmented in epileptic patients and in animal models of epilepsy (Mathern et al., 1998; Steffens et al., 2005). A decrease of PSD-95 scaffolding protein is supposedly responsible for a higher mobility of glutamate receptors and an elevation of extra-synaptic NMDARs, which may be relevant for the pathophysiology of epilepsy (Frasca et al., 2011; Gladding and Raymond, 2011; Parsons and Raymond, 2014). The location of NMDARs makes a significant functional difference (Hardingham and Bading, 2010; Bading, 2017). The subunit GluN1 is highly mobile and ligand interactions strongly influence the conformation of the receptor (Zhu et al., 2013). Overall, these results suggest that LRP1 intervenes in the uptake and recycling of glutamate receptors and thereby tunes the sensitivity of neuronal responses (Bading, 2017). However, the resulting changes in network activity are not sufficient to elicit seizures. Because the elimination of *Lrp1* in radial glia results in a deletion in both neurons and glia, the glial lineages also have to be considered with regard to the severe phenotype of the *Emx1-Cre::Lrp1* ^*fl/fl*^ knockout mutant.

### 1.7 Modulation of glutamate signalling by tPA-LRP1 interactions

Interestingly, the prominent LRP1 ligand tPA interacts with the GluN1 N-terminal domain and can elicit cytotoxicity (Nicole et al., 2001; Samson et al., 2008; Varangot et al., 2023). This notion has been elaborated further in that tPA interaction with GluN1 promotes the extra-synaptic diffusion of NMDARs without increasing the membrane located pool of the receptor (Lesept et al., 2016). The serine protease tPA belongs to the chymotrypsin family and consists of a heavy A-chain and a light B-chain. The A-chain contains a finger domain that mediates binding to fibrin and the membrane receptors Annexin II and LRP1 (Kagitani et al., 1985; Bu et al., 1992; Hajjar et al., 1994). Further structural motifs include an EGF-like domain that interacts with the EGFR and Kringle 1 and 2 domains, where the latter binds to NMDAR (Correa et al., 2011; Lesept et al., 2016). tPA is released as single chain and processed to its active form by plasmin or kallikrein (Leys et al., 2016). High concentrations of tPA can exert neurotoxic effects by stimulating the NMDAR (Bertrand et al., 2015; Chevilley et al., 2015; Wu et al., 2017). tPA has been implicated in a range of CNS pathologies, including Alzheimer disease and psychotic episodes (Fabbro and Seeds, 2009; Hoirisch-Clapauch and Nardi, 2015) and contributes to the integrity of the BBB (Polavarapu et al., 2007; Fredriksson et al., 2015; Stevenson et al., 2022). tPA is expressed in the CNS by excitatory pyramidal glutamatergic neurons, somatostatin expressing neurons of the hippocampus, and astrocytes (Fredriksson et al., 2015; Hebert et al., 2016; Louessard et al., 2016; Stevenson and Lawrence, 2018). The release of tPA affects synaptic activity and acts as a homeostatic regulator of the postsynaptic response, in dependence of activity and Ca^2+^ concentration (Wu et al., 2015; Jeanneret et al., 2016; Yepes, 2016). In the *Emx1-Cre::Lrp1* ^*fl/fl*^ mice no salient deviations of tPA concentrations compared to the control were observed (Bres et al., 2020). The exact impact of tPA on seizure generation and epilepsy progression is, however, manifold (Tan et al., 2012). tPA possesses a plethora of functions that are vital for CNS homeostasis (Hebert et al., 2016; Briens et al., 2017; Stevenson et al., 2022). tPA is highly expressed in brain regions undergoing cellular migration and mice deficient for tPA show an increased number of granule neurons that migrate in the molecular layer of the cerebellum (Seeds et al., 1999). tPA influences cortical neuron differentiation (Lee et al., 2014) and is implicated in proper radial glia maturation and neuron migration via interaction with the NMDAR expressed by radial glia (Pasquet et al., 2019). With regard to signaling, it is known that tPA interaction with LRP1 is important for the generation of late LTP in the hippocampus (Madani et al., 1999; Zhuo et al., 2000; Makarova et al., 2003; Wiera and Mozrzymas, 2015). tPA can also modulate NMDAR related signaling via LRP1 and thereby act in a neuroprotective or neurotoxic manner (Nicole et al., 2001; Jullienne et al., 2011; Ng et al., 2012; Parcq et al., 2012; Mantuano et al., 2013; Bertrand et al., 2015; Hedou et al., 2021). In the *Emx1-Cre::Lrp1* ^*fl/fl*^ mutant we observed by patch-clamp a hyperexcitability of pyramidal neurons in the hippocampus at P28 which was characterized by decreased action potential thresholds and increased action potential firing rates. This phenotype correlated with a decrease of PSD-95 and an elevated surface expression of the GluN1 subunit of the NMDAR (Bres et al., 2020).

### 1.8 Expression of LRP1 in the astrocyte lineage

LRP1 is expressed by neurons, but also by glial cells, namely, astrocytes and oligodendrocytes (Auderset et al., 2016a). The astrocytes play an important role for synapse formation, synaptic signaling and plasticity, which has led to the concept of the tripartite synapse (Araque et al., 1999; Perea et al., 2009; Faissner et al., 2010; Farhy-Tselnicker and Allen, 2018; Verkhratsky and Nedergaard, 2018). In this perspective, the astrocyte can intervene in synaptic signaling, modulates synaptic activity and has been attributed a role in the progression of epilepsy (Vezzani et al., 2022). For example, astrocytes express glutamate transporters Eaat1 (GLT1) and Eaat2 (GLAST) that serve glutamate clearance at the active excitatory synapse (Danbolt, 2001). This function can be altered in the case of epilepsy (Seifert et al., 2006; Steinhäuser and Seifert, 2012). Therefore, the astrocyte lineage was examined in more detail in the *Emx1-Cre::Lrp1* ^*fl/fl*^ mutant. When Lrp1 was deleted from NSPCs *in vitro*, an augmented production of GFAP-expressing astrocytes was detected in differentiation assays (Safina et al., 2016). Also the *Emx1-Cre::Lrp1* ^*fl/fl*^ mutant displayed a significant increase of GFAP, yet at P28, paralleling the emergence of severe seizures. These were also reflected by a strong increase of c-fos-positive neurons in the epileptic CNS (Bres et al., 2020). This raised the question whether the glutamate transport system was changed, also in light of the fact that GLT-1 is responsible for up to 90% of glutamate uptake in the synaptic region and has been implicated in epilepsy (Kong et al., 2012; Hanson et al., 2015; Petr et al., 2015; Hubbard et al., 2016; Hotz et al., 2022). Small variations of transporter expression were detected at earlier developmental stages, but not at P14 or later, when the seizure activity progresses. Therefore, tampered glutamate transport did not seem to be the major cause of seizure in the *Emx1-Cre::Lrp1* ^*fl/fl*^ mutant (Bres et al., 2020). It is known that tPA is co-released with glutamate at the glutamatergic synapse and taken up by neighboring astrocytes (Cassé et al., 2012). Therefore, a deletion of Lrp1 from the astrocyte membrane should reduce the clearance of tPA at active synapses. When the Lrp1-deficient astrocytes were tested the ability for tPA uptake was found strongly reduced (Bres and Faissner, 2019a; Bres et al., 2020). This led to the concept that the *Emx1-Cre::Lrp1* ^*fl/fl*^ mutant is characterized by an elevation of NMDARs, reduced clearance of tPA and transiently compromised glutamate clearance that result in a disturbed tPA-NMDAR signaling axis. This may contribute to or even cause the seizure phenotype that represents a salient feature of the mutant (Bres et al., 2020).

This idea was followed further by the generation of a conditional mutant where *Lrp1* was deleted exclusively from the astrocyte lineage (Romeo et al., 2020). To this end, the floxed reporter mouse line *GLAST-CreER* ^*T2*^ *::Rosa26* ^*fl/fl*^ was mated with the *LRP1* ^*fl/fl*^ mice to generate a triple-transgenic line that expresses the Tamoxifen-inducible Cre-recombinase under the control of the astroglial GLAST-promoter (Rohlmann et al., 1996; Mori et al., 2006; Madisen et al., 2010; Romeo et al., 2020). The reporter tdTomato allowed for tracing of the recombined cells (Table 1). The recombination and deletion of *Lrp1* could successfully be induced by addition of Tamoxifen to cultivated astrocytes. When the astrocytes obtained from the mutant were co-cultivated with hippocampal neurons in an *in vitro* coculture model (Geissler and Faissner, 2012; Gottschling et al., 2016), LRP1-deficient astrocytes caused changes in synaptogenesis and a decrease in synaptic activity in hippocampal neuronal networks. This was accompanied by a change of cytokine patterns released by the astrocytes in the coculture setting (Romeo et al., 2020). For further characterization, the recombination and deletion of *Lrp1* was induced in astrocytes *in vivo*, at early postnatal stages. In these mutants, the maturation of astrocytes appeared significantly retarded compared to controls. This resulted in a reduced neuronal activity *in vivo*, as monitored by the detection of the immediate early gene c-Fos in hippocampal neurons at P21 (Romeo et al., 2021). In accordance with this observation, a recent investigation has pointed out that the genetic knockout of APOE resulted in a decrease of lipid-accumulating reactive astrocytes and reduced seizures in a mouse model of temporal lobe epilepsy (TLE) (Chen et al., 2023). Lipid accumulation in astrocytes thus emerges as a risk factor for seizures and Alzheimer disease (Tcw et al., 2022; Chen et al., 2023). The maturation of astrocytes depends among others on the activation of the Ras/Raf/MEK/Erk signaling pathway (Franzdóttir et al., 2009; Li et al., 2012). This pathway can be activated by a complex of LRP1 with the PDGFβ-receptor (Boucher et al., 2002; Loukinova et al., 2002). Interestingly, the amount of activated Erk was significantly reduced in the LRP1-deficient *GLAST-CreER* ^*T2*^ *::Rosa26* ^*fl/fl*^ *::Lrp1* ^*fl/fl*^ mouse line at P21, consistent with a delay in astrocyte maturation. Cognitive abilities and motor coordination were also examined in the *GLAST-CreER* ^*T2*^ *::Rosa26* ^*fl/fl*^ *::Lrp1* ^*fl/fl*^ knockout mouse line and found unaltered. In conclusion, the conditional elimination of *Lrp1* from the astrocyte lineage revealed a link to astrocyte maturation. In contrast to the *Emx1-Cre::Lrp1* ^*fl/fl*^ mouse line, however, neither hyperexcitability nor seizures were observed in this model (Romeo et al., 2020; Romeo et al., 2021). This indicates that probably the LRP1 deficiency both in astrocytes and in neurons synergizes to produce the epilepsy phenotype (Bres et al., 2020).

It is of interest here to consider further approaches to elucidate LRP1 functions in astrocytes. The LRP1 and the LRP2 receptor are discussed as important modulators in the context of the Alzheimer disease because they contribute to the clearance of amyloid-beta (Aβ) peptide (Shinohara et al., 2017; Cai et al., 2018; Tachibana et al., 2019; Knopman et al., 2021). The selective knock-out of *Lrp1* in astrocytes results in an accumulation of Aβ-peptide in the CNS and exacerbates disease development in APP/PS1 mice, an established model of Alzheimer disease (Kanekiyo et al., 2013; Liu et al., 2017b). This underlines the importance of the LRP1-based clearance system in pathology, an aspect that has recently been emphasized by the report that LRP1 plays also a pivotal role in the clearance of pathogenic Tau-protein (Fearon and Lynch, 2020; Rauch et al., 2020) and of α-synuclein (Chen et al., 2022). However, also in this approach the removal of *Lrp1* from the astrocyte lineage did not result in an hyperexcitability phenotype.

### 1.9 Significance of LRP1 in the oligodendrocyte lineage and in myelin

The ablation of *Lrp1* from NSPCs *in vitro* also resulted in the impaired maturation of oligodendrocytes (Safina et al., 2016). This may reflect the fact that oligodendrocyte maturation heavily relies on the supply of cholesterol (Saher et al., 2005). Myelin malformation may cause both motor deficits and hyperexcitability in neural tissues (Nave and Werner, 2014; Nave and Werner, 2021). Therefore, potential roles of LRP1 for myelin formation were examined in more detail. It is known that LRP1 is expressed by young oligodendrocytes and oligodendrocyte precursors and rapidly lost upon differentiation (Zhang et al., 2014; Auderset et al., 2016a). An important task of LRP1 with reference to myelin is the clearance of debris in the context of lesion (Gaultier et al., 2009; Fernandez-Castaneda et al., 2013; May, 2013; Sharp et al., 2023). Furthermore, LRP1 can bind the myelin-associated glycoprotein (MAG) and expose the inhibitor to neurites (Stiles et al., 2013). Oligodendrocytes are generated in three distinct waves and colonize the developing nervous system through a migratory process (Rowitch and Kriegstein, 2010). Therefore, LRP1 may be of significance for myelin formation during the early developmental stages of oligodendrocyte precursor cell (OPC) formation. In order to explore the role of LRP1 for OPCs, a novel triple transgenic mouse line was elaborated (Table 1). To this end, a triple transgenic mouse model was generated that expressed the inducible Cre-recombinase under the control of the NG2-promoter (Huang et al., 2014). The resulting *NG2-CreER* ^*T2*^ *::R26eGFP::Lrp1* ^*fl/fl*^ line enabled the induced recombination and removal of LRP1 from NG2-positive cells. The recombined cells would furthermore express the eGFP reporter, so that the LRP1 deficient cells could efficiently be tracked *in vivo* and *in vitro* (Schafer et al., 2019). The knockout of *Lrp1* was induced three and 4 days after birth and the resulting mice were examined at P7, P14, P21, P28, P42 and P56. The development of oligodendrocytes and myelin was monitored in the corpus callosum, a strongly myelinated structure of the CNS. The recombination and removal of LRP1 in the oligodendrocyte lineage proved successful. However, no significant alterations of myelin were observed in the mutant in comparison to the control, and no apparent motor deficits could be recorded. In contrast, when the fluorescent marker protein eGFP was set in relation to the Olig2 marker of the oligodendrocyte lineage it appeared that the recombined, fluorescent Olig2-positive cells dramatically decreased during the postnatal period. This was interpreted as indicating that the LRP1-deficient, reporter expressing and Olig2-positive double fluorescent OPCs progressively were eliminated from the system and presumably replaced by non-recombined NG2-and LRP1-positive OPCs (Zhu et al., 2008; Dimou and Gallo, 2015; Schafer et al., 2019). This result suggests a stronger vulnerability of the LRP1-deleted OPCs, in agreement with the previous findings reporting reduced numbers of oligodendrocytes obtained from *Lrp1*-deficient recombined NSPCs (Safina et al., 2016). Accordingly, *in vitro* studies using immuno-panned OPCs only recovered a small minority of <3% LRP1-negative, fluorescent recombined cells, much less than expected, which could be explained by the increased vulnerability of the LRP1-deleted OPCs (Schafer et al., 2019). In another approach where LRP1 was deleted from the oligodendrocytes using *Olig2-Cre::Lrp1* ^*fl/fl*^ mice the mutants showed hypomyelination of the optic nerve and a clear decrease of differentiated oligodendrocytes. Furthermore, in both *CAG-CreER* ^*TM*^ *::Lrp1* ^*fl/fl*^ and *PDGFRa-CreER* ^*TM*^ *::Lrp1* ^*fl/fl*^ the ability to repair myelin was restricted compared to the wild-type (Lin et al., 2017). This phenotype was correlated with a decrease of peroxisomal biogenesis factor-2 and a reduced number of peroxisomes in oligodendrocyte processes. The deficits could be corrected by a combined treatment with cholesterol and pioglitazone, a drug that promotes peroxisome proliferation (Lin et al., 2017). Recently, a study using the *PDGFRa-CreER* ^*TM*^ *::Rosa26-YFP::Lrp1* ^*flflf*^ line addressed the relevance of LRP1 in adult CNS myelin, with focus on the corpus callosum (Table 1). Upon Tamoxifen treatment, *Lrp1* could successfully be eliminated from oligodendrocytes in the adult CNS, which, surprisingly, resulted in an increase of OPC proliferation and of the number of new oligodendrocytes added to the adult brain. Furthermore, the number of OPCs recruited upon Cuprizone feeding was also augmented. This correlated with an heightened proportion of OPCs that differentiated *in vitro* (Auderset et al., 2020). Considering the contrast of these results with the studies of LRP1 ablation in the oligodendrocytes during the postnatal period, the authors hypothesize that postnatal and adult oligodendrocytes may have different properties and requirements (Auderset et al., 2020). Of note, in neither of the reported lineage-restricted eliminations of LRP1 from oligodendrocytes and myelin motor deficits, let alone hyperexcitability or seizures could be detected. Thus, the removal of LRP1 from the radial glia stem cell has a stronger impact than the one obtained by elimination of LRP1 from individual neural lineages (Table 1).

### 1.10 Conclusion and outlook

The receptor protein LRP1 interacts with more than 40 different ligands and plays a pivotal role for the uptake and clearance of a large variety of molecules. It has so far been considered in the context of disease, in particular Alzheimer disease in view of its role in the clearance of Aβ-peptide (Storck et al., 2016; Eggert et al., 2018; Van Gool et al., 2019). In view of its importance it is not surprising that the constitutive knock-out of *Lrp1* is lethal at early embryonic stages (Herz et al., 1992). While the major emphasis in studying LRP1 has been oriented towards its role in the homeostasis of tissues and in protecting the CNS against the accumulation of pathogenic metabolic products, we have discovered a prominent role in the stem cell compartment during early development (Hennen et al., 2013; Safina et al., 2016). Several animal models have been constructed to decipher the functions of LRP1 in distinct cellular lineages of the developing CNS (Table 1). It appears that by far the most dramatic phenotype was obtained when *Lrp1* was deleted from the cortical radial glia compartment using the *Emx1* promoter as driver of Cre recombinase (Bres et al., 2020). Neither the knockout in neurons (Liu et al., 2010; Maier et al., 2013), nor the knockout in oligodendrocytes (Lin et al., 2017; Schafer et al., 2019; Auderset et al., 2020) or astrocytes (Liu et al., 2017b; Romeo et al., 2020; Romeo et al., 2021) yielded a phenotype of comparable strength. This opens the perspective that seizures may be rooted in a neurodevelopmental deficit affecting the neural stem cell compartment. The identification of the genes and pathways involved may in the future benefit from the application of genomic and transcriptomic strategies to the mouse model (Busch et al., 2022; Lariviere et al., 2022; Pai et al., 2022; Assis-Mendonca et al., 2023; Chung et al., 2023; Huang et al., 2023).

## Statement of significance

The low-density lipoprotein related protein receptor 1 (LRP1, also known as CD91) is a transmembrane receptor that interacts with more than 40 known ligands. In the CNS, it has primarily been studied as a receptor and clearance agent of pathogenic factors such as Abeta-peptide and, lately, Tau protein that is relevant for tissue homeostasis and protection against neurodegenerative processes. Recently, we have found that LRP1 is expressed in the neural stem cell compartment and regulates the development of glial lineages. When LRP1 was eliminated from cortical radial glia a strong phenotype with motor coordination deficits and severe seizures resulted. In the present review I discuss the attempts that have been made to understand the link of LRP1 expression in glial lineages to the generation of epilepsy, a burdening human disease. In particular, several conditional mouse lines with lineage-specific ablation of LRP1 have been constructed and described. Taken together, the studies strongly suggest that cooperative effects between neurons and glia are responsible for the phenotype. These observations emphasize LRP1 as an important regulator of tissue homeostasis and highlight the significance of the neural stem cell compartment for neurodevelopmental disorders and CNS pathology.
